# To vax or not to vax: Predictors of anti-vax attitudes and COVID-19 vaccine hesitancy prior to widespread vaccine availability

**DOI:** 10.1371/journal.pone.0264019

**Published:** 2022-02-15

**Authors:** Hannah A. Roberts, D. Angus Clark, Claire Kalina, Carter Sherman, Sarah Brislin, Mary M. Heitzeg, Brian M. Hicks

**Affiliations:** 1 Department of Psychology and Neuroscience, Temple University, Philadelphia, PA, United States of America; 2 Department of Psychiatry, University of Michigan, Ann Arbor, MI, United States of America; 3 Department of Psychology, Virginia Commonwealth University, Richmond, VA, United States of America; University of Haifa, ISRAEL

## Abstract

The novel coronavirus (COVID-19) is a highly contagious disease responsible for millions of deaths worldwide. Effective vaccines against COVID-19 are now available, however, an extreme form of vaccine hesitancy known as *anti-vax* attitudes challenge vaccine acceptance and distribution efforts. To understand these anti-vax attitudes and their associated psychological characteristics, we examined several predictors of vaccine hesitancy for COVID-19 and anti-vax attitudes generally. We surveyed 1004 adults (M = 47.0 years, SD = 17.1 years, range 18–98 years) in September-October 2020 across the United States (51% female, 49% male; 76.5% White, 23.5% non-White), prior to widespread availability of the COVID-19 vaccines. Attitudes toward vaccinations were influenced by a variety of factors, especially political attitudes. We should therefore anticipate and attempt to mitigate these challenges to achieving widespread vaccination to reduce the spread of COVID-19 and other communicable diseases.

## Introduction

The Centers for Disease Control (CDC) reports that vaccination is the single most effective tool in preventing infectious disease [1]. The societal benefits of vaccination are achieved via herd immunity, a process whereby transmission of an infectious disease is unlikely if enough members of the population achieve immunity from the disease. As such, vaccines play a critical role in suppressing the spread of contagious diseases and ending epidemics/pandemics.

In March 2020, the novel coronavirus (COVID-19) was declared a pandemic and has since infected over 393 million people worldwide and claimed more than 5.7 million lives, with the U.S. accounting for almost 15% of these deaths [2, 3]. Besides posing an incredible health concern to the American population, the financial effects of COVID-19 burden the U.S. economy. For example, 7.7 million Americans were facing unemployment due to the COVID-19 pandemic in June 2020. Consequently, those individuals and their dependents lost health insurance coverage, resulting in a total of 14.6 million newly uninsured Americans and led to a large subset of the US population unable to pay rent and purchase necessities [4, 5].

As of February 2022, 64% of Americans were fully vaccinated against COVID-19 [3]. However, a major roadblock in administering widespread COVID-19 vaccines to the American public is hesitancy towards the COVID-19 vaccine, as well as skepticism to vaccination in general called *anti-vax* attitudes. The World Health Organization (WHO) has identified anti-vax attitudes as a major threat to global health [6, 7]. Anti-vax attitudes can be considered part of a cultural debate, rather than a scientific one, making them particularly resistant to scientific and medical consensus [8]. People with anti-vax beliefs have the devasting potential to increase the spread of infectious disease despite the availability of effective vaccines, and thus it is important to understand the demographic, psychological, and social correlates of this group to improve targeted public health interventions.

### The far left and right converge to fight vaccination

Vaccine hesitancy has existed since the introduction of vaccines. In 1796, Edward Jenner developed the first vaccine against a communicable illness (smallpox) effectively preventing infection and the spread of the disease [9]. Shortly after, several organizations including the Anti-Vaccination League protested the dissemination of the vaccine due to safety concerns, and encouraged homeopathic solutions instead [10]. This discourse continued modestly throughout the early 20th century but was reignited in the U.S. as part of the 1970’s “hippie” counter-culture. In 1998, a since retracted publication in a well-respected medical journal suggested a link between the mumps, measles, and rubella (MMR) vaccine and autism [11–13]. As a result of this publication and the discourse surrounding it, anti-vax beliefs gained considerable traction within liberal, “neo-hippie” communities, which were often found in affluent and mostly White enclaves. These communities endorsed the idea that a natural and organic lifestyle would provide a sufficient immune response to combat infectious diseases, even more so than vaccines [14].

While the anti-vax movement gained ground on the political left in 1998, a sizable far-right political group more recently joined the anti-vax movement, citing mistrust of the government and “Big Pharma” (i.e., the American medical and pharmaceutical establishment) as their main reasons for vaccine avoidance [15]. Prior to the COVID-19 pandemic, former Republican President Donald Trump aligned himself with the right-wing of the anti-vax movement, which created a mixed political message when the focus of his administration’s pandemic response to COVID-19 was to accelerate vaccine development [16]. Trump also consistently attempted to play down the severity of the health risks associated with COVID-19 via statements that were often partisan in nature. Consequently, it is likely that former President Trump’s messages contributed to the beliefs among conservative Americans that the COVID-19 pandemic is not a serious health concern and vaccination is not an important and effective strategy for preventing the infection and spread of the disease [17, 18]. This can be illustrated by a survey conducted in June 2020 which found that people who intended to vote for Trump in the 2020 presidential election were less willing to receive a COVID-19 vaccine when it became available [19]. Thus, while far-left and far-right groups cite different reasons for vaccine refusal, their behaviors manifest in the same way (i.e., vaccine refusal), contributing to a major global health concern.

### Correlates of anti-vax attitudes: Demographics, psychological characteristics, political attitudes, and COVID-19 experiences

Our goal was to conduct an examination of the correlates of anti-vax attitudes in general and COVID-19 vaccination hesitancy specifically. Besides socio-political attitudes, it is important to understand how demographic characteristics are associated with vaccination hesitancy. Recent studies of COVID-19 vaccine hesitancy in the UK and the U.S. found that Black and other non-White members of the population were less willing to receive a COVID-19 vaccine [19, 20]. This trend has been observed with other communicable illnesses, including MMR and the annual flu vaccine [20, 21]. Similarly, in most U.S. states, Black and Hispanic Americans are receiving COVID-19 vaccinations at lower rates than White American [22]. These trends, in part, may be explained by a history of racial barriers and injustices in the American medical establishment. For example, people of color report more frequent negative experiences with healthcare providers and lower rates of health insurance coverage than White Americans [23].

Low levels of educational attainment have been generally associated with greater vaccine hesitancy [24]. Research on hesitancy in yearly flu vaccine uptake indicates that motivations for hesitancy differ across the education spectrum, with more educated people having greater skepticism of the scientific mechanisms of the medicine and its efficacy and safety, while less educated people may decline vaccination due to lack of information [24]. The associations between vaccine hesitancy and income are less clear [25]. Studies examining the role of income in willingness to receive the COVID-19 vaccine have found associations between lower income and COVID-19 hesitancy as well as null effects [19, 20]. Makarovs and Achterberg (2017) suggest that income may be best understood in the context of overall socioeconomic status, which combines both income and education, making a test of the relative predictive power of income on vaccine hesitancy novel [24].

In research exploring COVID-19 vaccine uptake, women reported greater vaccine hesitancy than men [19, 20]. Women often bear most of the responsibility for family health care, and so it may be that women are generally more aware of and concerned about negative side effects of vaccinations [19]. There are mixed results for age on COVID-19 vaccine hesitancy, with studies showing both greater hesitancy in young people and no effect of age [20, 26]. Historically, there has been limited research on the association between sex, age, and vaccine hesitancy.

The role of broad psychological characteristics such as personality, mental health, and substance use have been understudied as a correlates of anti-vax attitudes. However, they play an important role in health behaviors and may therefore contribute to anti-vax attitudes and hesitancy. For example, personality traits such as low conscientiousness and neuroticism as well as depression, smoking, and heavy alcohol use are all associated with poorer physical health and health-related behaviors in general, and so may contribute to anti-vax attitudes or slow vaccine uptake [27–31].

Liberal and conservative social and political attitudes are likely to have strong associations with anti-vax attitudes. Given that Donald Trump was the standing president during when the COVID-19 pandemic began, we examined Trump job approval as it related to COVID-19 vaccine hesitancy and anti-vax attitudes generally. Additionally, the COVID-19 pandemic has been characterized by substantial misinformation or “fake news” regarding origin of the virus, contents of the COVID-19 vaccine, etc., which has been propagated through social media, now the main source of news among a large proportion of Americans [32, 33]. Anti-vax groups of both the left and right variety also utilize social media to amplify their message and recruit new members. Consequently, we tested whether social media use—especially problematic use associated with negative consequences and an inability to control use—made an additional contribution to anti-vax attitudes and COVID-19 vaccine hesitancy over and above other psychological characteristics and behaviors.

Finally, it is likely that people’s individual experiences during the COVID-19 pandemic will relate to their attitudes about vaccination over and above more general characteristics like demographics, personality, mental health, and political attitudes. Therefore, we included several measures related to COVID-19 experiences, emotions, and behaviors including COVID-19 related worry and stress and negative impacts associated with the pandemic such as illness to self or relatives and friends, financial stress, restriction of activities, etc. We also included people’s adherence to recommendations for safety behaviors and mitigation strategies (e.g., mask-wearing and social distancing) and their attitudes about government mandates and restrictions.

### Current study

We examined the role of political attitudes, personality, mental health, and substance use on anti-vax attitudes and vaccine hesitancy. In consideration of the current political landscape, we measured alignment with former President Trump’s ideology regarding COVID-19 vaccine hesitancy and broad anti-vax attitudes. Also, as social media has been an important way through which false information regarding COVID-19 has been propagated, we examined the impact of social media use on both COVID-19 vaccine hesitancy and broad anti-vax attitudes. Finally, we explored the impact of several proximal social and environmental factors related to the COVID-19 pandemic itself; negative impacts associated with the pandemic, COVID-19 related worry and stress, adherence to recommendations for safety behaviors and mitigation strategies, and attitudes about government mandates and restrictions.

We hypothesized that more conservative and less liberal sociopolitical attitudes, greater approval of Trump, less conscientiousness and more neuroticism, greater depressive symptoms, greater substance use, and social media use and problematic use would be associated with greater anti-vax attitudes and COVID-19 vaccine hesitancy. We also predicted that people who had experienced more COVID-19 related stress/worry, more negative impacts, and were more adherent to mitigation strategies and had higher approval of government restrictions would endorse less anti-vax attitudes in general and less COVID-19 vaccine hesitancy.

## Methods

### Sample ascertainment

Data were collected from September 24 through October 25, 2020, using an actively managed, double-opt-in research panel using Qualtrics XM survey software. Recruitment was designed to ascertain a sample consistent with major demographic characteristics of the United States general population for age (12.8% 18–24; 17.7% 25–34; 16.7% 35–44; 17.7% 45–54; 16.4% 55–64; 18.8% +65), gender (51% female; 49% male), and race/ethnicity (61.9% non-Hispanic White; 12.3% non-Hispanic Black; 17.4% Hispanic; 5.3% Asian; 3.2% Other). Quotas were created for each variable and monitored while the survey was in the field. Respondents were recruited using a dashboard-style web page on the Qualtrics website and cellphone app where participants see a list of surveys that they have the option to participate in. Recruitment was also conducted through emails sent to established panel members within the Qualtrics database. In all recruitment methods, potential participants received information on the estimated length of the survey and compensation for completing it. Specific details about the survey content were not available until the participants opted-in to avoid self-selection bias. Upon opting into the study, participants read and provided an electronic signature on a consent form containing an overview of the survey contents. Participation was voluntary and anonymous as no individually identifying information was collected. Upon completion, participants were awarded credits by Qualtrics which they could cash out or use to purchase gift cards. Contact information for the research team was provided in case participants had questions about the survey. The University of Michigan Medical School Institutional Review Board (IRB) reviewed all study protocols.

The survey was completed by 1024 respondents. Data were manually checked, and 20 respondents were excluded due to inconsistent and illogical responses, resulting in a final sample size of 1004 respondents (487 men, 512 women, and 5 that reported non-binary gender). Examples of excluded responses include those who failed item-level quality control checks and those who consistently (>75%) chose only one response option, e.g., all “2”s. Single measures were excluded on a case-by-case basis if all other responses from that participant were within a plausible range of values. The final sample included responses from participants aged 18 to 98 years old (M = 46.5 years, SD = 17.1). The median response time for completing the survey was 28.1 minutes.

### Demographics

The demographic characteristics of the sample are reported in Table 1, which were similar to those reported in the 2019 American Community Survey (ACS) 1-year estimates conducted by the US Census Bureau, though some differences occurred in education due to a lack of quota for this variable. The gender split for our sample was 51.1% female, 48.9% male (51.3% female, 48.7% male in US population ages 18 years and older). The age distribution for the survey sample was similar to the US general population with the proportion of the sample in 10-year age bands differing by 0% to 1.1% to that of the ACS. Mean educational level was higher than the US general population due to lower rates of people with a high school diploma or less (17.3% sample versus 39.1% US population ages 18 years and older) and higher rates of people with a bachelor (34.7% sample versus 19.3% US population) and graduate degree (22.3% sample versus 11.3% US population). Mean household income was also higher than the US general population with lower incomes (< $50,000) underrepresented (34.2% sample vs 38.4% US households) while middle incomes ($50,000 to $100,000; 33.2% sample vs 30.2% US households) and higher incomes (> $100,000; 32.6% sample vs 31.4% US households) were somewhat overrepresented. Hispanic ethnicity (17.7% sample vs 16.8% US population 18 years and over) and racial representation were similar to the US census (White 76.2% sample, 74.6% US population; Black or African American 14.3% sample vs 13.0% US population; Asian 6.0% sample vs 4.9% US population). Other races represented in the sample but not included here are American Indian, Alaska Native, Native Hawaiian and Pacific Islander. In terms of political party affiliation, persons affiliated with the Democratic party were overrepresented (39.0% vs 33.0%), persons affiliated with the Republican party were slightly underrepresented (26.5% vs 29.0%) and persons identified as independent or not registered with a political party were also slightly underrepresented (31.5% vs 34.0%) relative to the general electorate [34]. Three percent of the sample identified with another political party (Libertarian, Green, Constitution, Independent, or other).

**Table 1:** Demographics.

	Total (*N* = 1004)	Men (*n* = 491)	Women (*n* = 513)
	% (*n*)	% (*n*)	% (*n*)
**Age (years)**			
18–29	17.9 (179)	8.8 (43)	26.5 (136)
30–39	19.8 (199)	19.3 (95)	20.3 (104)
40–49	16.7 (168)	17.3 (85)	16.2 (83)
50–59	16.2 (163)	18.1 (89)	14.4 (74)
60–69	17.6 (177)	19.8 (97)	15.6 (80)
70+	11.2 (112)	15.9 (78)	6.6 (34)
**Race**			
White	76.1 (764)	79.0 (388)	73.3 (376)
Black	14.3 (144)	10.8 (53)	17.7 (91)
Other race	9.6 (96)	10.2 (50)	9.0 (46)
Hispanic- any race	17.7 (178)	16.5 (81)	18.9 (97)
**Education**			
No high school degree	3.1 (31)	1.8 (9)	4.3 (22)
High school diploma	14.2 (143)	9.4 (46)	18.9 (97)
Some college	25.7 (258)	20.2 (99)	31.0 (159)
Bachelor’s degree	34.7 (348)	38.3 (188)	31.2 (160)
Master’s degree	17.7 (178)	23.2 (114)	12.5 (64)
Doctorate	4.6 (46)	7.1 (35)	2.1 (11)
**Annual Household Income**			
$0–9,999	5.0 (50)	3.3 (16)	6.6 (34)
$10,000-$24,999	10.4 (104)	6.7 (33)	13.8 (71)
$25,000–49,999	18.8 (189)	12 (59)	25.3 (130)
$50,000–74,999	17.0 (171)	17.5 (86)	16.6 (85)
$75,000–99,999	16.2 (163)	19.1 (94)	13.5 (69)
$100,000–149,999	18.3 (184)	22.6 (111)	14.2 (73)
$150,000+	14.2 (143)	18.7 (92)	9.9 (51)

*Note*: “Other race” includes the following responses: Asian or Asian American (5.8%, n = 58, American Indian or Alaska Native (.60%, n = 6), Native Hawaiian or Pacific Islander (.10%, n = 1), Mixed Race (1.5%, n = 15) and Don’t know/Missing (1.6%, n = 16).

### Measures

#### Outcome variables

*Anti-vax scale*. Participants responded to six questions assessing general support for vaccinations (strongly disagree = 1, disagree = 2, somewhat disagree = 3, somewhat agree = 4, agree = 5, strongly agree = 6; M = 17.11) [35]. Item content and psychometric properties are reported in Table 2. The six items exhibited good to excellent discrimination parameters in graded item response models. The mean discrimination parameter was *a* = 2.52 (range .94 to 6.29) and the mean standardized factor loading was λ = .72 (range .48 to .97). The marginal reliability for the scale was .86 and internal consistency (Cronbach’s α) was .79. Graded response models were estimated using flexMIRT version 3.6 with full information maximum likelihood estimation [36].

**Table 2:** Anti-vax scale items and psychometric properties.

	% Endorsement for each response option								
Item	Strongly Disagree	Disagree	Somewhat Disagree	Somewhat Agree	Agree	Strongly Agree	a	λ	α if item deleted
Vaccines are more dangerous than the disease they are trying to prevent.	43.0	18.6	16.5	10.1	6.3	5.5	6.29	.97	.72
Vaccines administered to children at young ages may cause them to become autistic.	40.1	19.1	14.8	11.6	7.5	7.0	3.35	.89	.73
When parents decide not to vaccinate, it puts their children and communities at risk. (r)	4.1	2.5	7.0	16.8	23.5	46.2	1.92	.75	.76
The government should require all parents to have their children vaccinated against contagious diseases. (r)	6.8	5.7	10.9	21.3	21.6	33.8	1.39	.63	.78
Parents understand their child’s medical needs better than anyone else.	12.6	14.1	21.2	22.4	17.1	12.6	1.20	.58	.78
I like to do my own research before agreeing to a vaccination.	7.0	8.7	12.4	28.9	23.7	19.3	.94	.48	.79

*Note*: r = reverse scored item; a = item discrimination parameter; λ = standardized factor loading; α = Cronbach’s alpha. Discrimination parameters and factor loadings were derived from graded item response models. The marginal reliability of the scale was .86 and Cronbach’s alpha for the scale was .79.

*COVID-19 vaccine hesitancy*. We assessed openness to receiving a vaccination for the COVID-19 virus with the single item “How likely are you to get a COVID-19 vaccine that has gone through the normal protocol for development (e.g., clinical trials, tests for safety and efficacy)?” *(very unlikely*, *unlikely*, *likely*, *very likely)*. A COVID-19 vaccination had not yet been released in any country at the time of assessment.

#### Predictor variables

*Demographics*. Demographic characteristics used in the prediction models included age, sex assigned at birth (male, female), race (White, non-White), annual household income, and educational attainment.

*Personality*. The Big Five Inventory-2 short form (30-items) was used to assess extraversion (sociability, assertiveness, energy level; α = .71), agreeableness (compassion, respectfulness, trust; α = .76), conscientiousness (organization, productiveness, responsibility, α = .78), negative emotionality (anxiety, depression, emotional volatility, α = .85), and open-mindedness (aesthetic sensitivity, intellectual curiosity, creative imagination, α = .68) [37].

*Mental health*. We used the general Depression scale (20-items; α = .93) from the Inventory of Depression and Anxiety Symptoms to provide a measure of overall depressive symptoms and dysphoria [38].

*Binge drinking*. We assessed alcohol use by asking participants to report how often they had engaged in binge drinking (i.e., five or more drinks on one occasion) in the past 30 days *(Never*, *once a month*, *2–3 times per month*, *once or twice per week*, *3–4 times per week*, *nearly every day*, *twice per day*, *three or more times per day*).

*Nicotine use*. Participants reported frequency of smoking cigarettes, using smokeless tobacco, or e-cigarettes (*Never*, *once or twice*, *occasionally but not regularly*, *regularly in the past*, *regularly now*). Response options *“regularly in the past”* and *“regularly now”* were coded the same for the analyses. The highest frequency reported among the three nicotine questions was used for the nicotine use variable.

*Social media use*. Participants indicated their use of a list of social media platforms. If they indicated the use of any social media platform, they were coded as a “1” on a binary social media use variable. If they indicated a response of “None”, they were coded as a “0”.

*Problematic social media use*. A 9-item scale (α = .88) was used to assess problematic social media use. Items were modeled on facets of behavioral addiction including overuse, functional impairment, using social media to reduce negative emotions, and attempts to cut down on use without success (e.g., “Spent a lot of time thinking about social media.”, “Used social media so much that it has had a negative impact on your job/studies.”).

*Liberal and conservative social attitudes*. We used the Right-Wing Authoritarianism (RWA; 22-items) scale to provide a measure of liberal and conservative political and social attitudes [39]. Ten items were worded in the direction of endorsement of more liberal attitudes (α = .88; e.g., “Everyone should have their own lifestyle, religious beliefs, and sexual preferences, even if it makes them different from everyone else.”) and 12 items were worded in the direction of more conservative and authoritarian attitudes (α = .92; e.g., “The only way our country can get through the crisis ahead is to get back to our traditional values, put some tough leaders in power, and silence the troublemakers spreading bad ideas.”). The liberalism and conservatism scales had a significant negative correlation (*r* = -.54), but still had sufficient unique variance to provide incremental predictive power relative to a single scale.

*Approval of President Trump*. We asked respondents to report their approval of President Trump’s job performance in general *(strongly approve*, *approve*, *somewhat approve*, *somewhat disapprove*, *disapprove*, *strongly disapprove)*.

*COVID-19 related safety behaviors and attitudes about government mandates and restrictions*. For safety behaviors, participants were asked how often they followed the “social distancing” or “shelter-in-place” restrictions put in place in your community in the past 3 months, and how often they wear a mask when in public *(never*, *seldom*, *sometimes*, *often*, *always)*. Participants were also asked three questions to assess attitudes about government responses to COVID-19 including the necessity of COVID-19 related restrictions (*very necessary*, *necessary*, *somewhat necessary*, *neither necessary or unnecessary*, *somewhat unnecessary*, *unnecessary*, *very unnecessary*), approval of their state government’s social distancing restrictions (*strongly approve*, *approve*, *somewhat approve*, *somewhat disapprove*, *disapprove*, *strongly disapprove*), and attitudes about the pace at which their community was lifting social distancing restrictions (*much too soon*, *somewhat too soon*, *about right*, *somewhat too late*, *much too late*). A composite variable of COVID-19 related safety behaviors and attitudes about government restrictions was calculated by taking the mean of the five items (α = .81).

*COVID-19 stress/worry*. Seven items from the COVID-19 Adolescent Symptom and Psychological Experience Questionnaire were used to assess COVID-19 related negative emotions during the past 30 days [40]. Participants reported their level of general stress and worry related to the COVID-19 pandemic, how stressful they have found the related restrictions on leaving home, disruptions to future plans, and uncertainty of the future related to the COVID-19 pandemic. Participants also reported their level of worry about becoming infected with COVID-19, their physical and mental health being impacted by COVID-19, and friends or family being infected with COVID-19 (α = .92).

*Negative impacts of the COVID-19 pandemic*. We assessed the number of negative impacts incurred due to the COVID-19 pandemic by providing participants with a multiple-response list of negative events including: *Self/loved one has the virus; having to stay home; not seeing friends in person; many people are dying because of the virus; not going to school/work; self/child participating in virtual education; spending more time with family; financial difficulties; concern about the health of self/loved ones; concern about the government; missing important events;* and a free-response option. A composite variable of negative impacts was calculated by taking the sum of all responses (0 = was not selected as a negative event, 1 = selected as a negative event).

### Data analytic strategy

**Fig 1:**
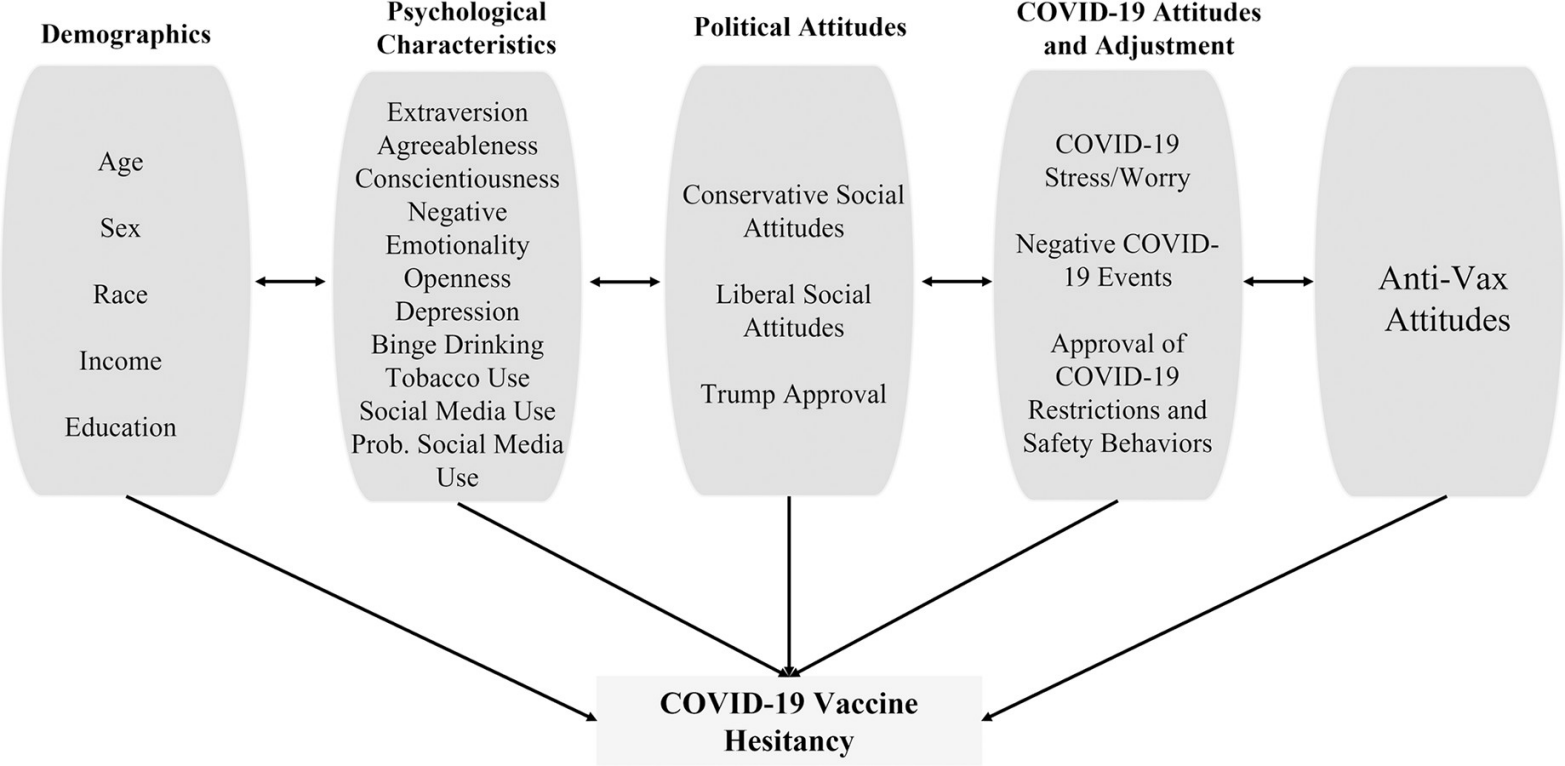
All predictors from COVID-19 vaccine hesitancy models.

Separate multiple regression models were fit to examine the extent to which predictor variables were associated with the anti-vax beliefs and hesitancy to receive a COVID-19 vaccine. Fig 1 illustrates the organization of the predictors into conceptually related blocks. Blocks were ordered in increasing conceptual proximity to the individual outcome (from right to left in Fig 1). Vaccine outcomes were first regressed on a block of demographic predictors that included: age, sex assigned at birth, White versus non-White race, income, and education. The second block of predictors included person-level psychological variables and behaviors including personality traits, depression, alcohol and nicotine use, social media use (Yes/No), and problematic social media use. The third block of predictors included social and political attitudes including conservative and liberal attitudes in general and the job approval of then President Donald Trump specifically. The fourth block captured behaviors and attitudes specific to the COVID-19 pandemic, including stress/worry about COVID-19, negative impacts incurred as a result of the COVID-19 pandemic, and frequency of following safety behaviors and attitudes about government restriction to mitigate the spread of COVID-19. Anti-vax beliefs were also included as a predictor of hesitancy to receive a COVID-19 vaccine after this fourth block.

All models were fit in Mplus version 8.5 using full information maximum likelihood estimation [41]. Confidence intervals were derived via nonparametric percentile bootstrapping (10,000 draws), which provides reliable assessments of parameter estimate precision across a variety of data conditions [42].

## Results

See https://osf.io/cqa6m/?view_only=659f436af1ce4049b2e1d01d8b540343 for full data set. Table 2 provides the endorsement rates for each anti-vax option. Based on these endorsement rates, about 40% of the sample strongly disagreed with anti-vax attitudes and about 60% of the sample either disagreed or strongly disagreed with anti-vax attitudes. About 4%-7% of the sample strongly agreed with anti-vax attitudes and about 10%-15% either agreed or strongly agreed with anti-vax attitudes. Table 3 provides the correlations between the six individual items that comprise the anti-vax scale. Table 4 provides the correlations among all predictors in both models.

**Table 3:** Anti-vax scale item correlations.

Item	1	2	3	4	5	6
1. Vaccines are more dangerous than the disease they are trying to prevent.	-					
2. Vaccines administered to children at young ages may cause them to become autistic.	.777	-				
3. When parents decide not to vaccinate, it puts their children and communities at risk. (r)	.486	.444	-			
4. The government should require all parents to have their children vaccinated against contagious diseases. (r)	.411	.324	.578	-		
5. Parents understand their child’s medical needs better than anyone else.	.431	.408	.256	.219	-	
6. I like to do my own research before agreeing to a vaccination.	.342	.334	.204	.222	.395	-

Note: All correlations are significant at the .001 level. Each successive regression model was associated with a significant increase in *R*^2^ at *p* < .001.

**Table 4:** Predictor and outcome correlations.

Item	1	2	3	4	5	6	7	8	9	10	11	12	13	14	15	16	17	18
1. Age	-																	
2. Income	**.152**	-																
3. Education	**.147**	**.447**	-															
4. Extraversion	**.157**	**.186**	.114	-														
5. Agreeableness	**.281**	-.016	-.068	**.241**	-													
6. Conscientiousness	**.440**	.076	**.134**	**.270**	**.503**	-												
7. Neg. Emotionality	**-.439**	**-.230**	**-.231**	**-.455**	**-.431**	**-.553**	-											
8. Openness	-.024	-.039	.019	**.314**	**.271**	**.270**	**-.250**	-										
9. Depression	**-.444**	**-.105**	**-.112**	**-.306**	**-.379**	**-.570**	**.635**	-.176	-									
10. Binge Drinking	**-.233**	**.097**	.037	-.028	**-.216**	**-.310**	**.133**	**-.160**	**.354**	-								
11. Smoking	**-.264**	-.009	**-.105**	-.032	**-.132**	**-.250**	**.212**	-.026	**.372**	.417	-							
12. SM Use	**-.321**	-.062	-.071	.026	.031	**-.178**	**.124**	.092	**.186**	**.144**	**.141**	-						
13. Prob. SM Use	**-.503**	-.054	-.026	-.047	**-.240**	**-.492**	**.352**	-.099	**.568**	**.442**	**.385**	**.445**	-					
14. Conservative Social Attitudes	.031	-.040	**-.096**	.004	-.039	-.007	-.010	**-.273**	.062	**.190**	**.120**	.065	**.151**	-				
15. Liberal Social Attitudes	-.063	**.089**	.060	-.033	.038	-.057	.046	**.181**	.036	-.040	.044	-.004	.027	**-.537**	-			
16. Trump Attitudes	-.034	.052	-.014	.030	**-.120**	-.057	.002	**-.165**	.031	**.190**	**.152**	.014	**.096**	**.589**	**-.448**	-		
17. COVID-19 Stress/Worry	**-.155**	.032	.046	-.088	-.104	**-.241**	**.325**	.015	**.520**	**.142**	**.142**	**.104**	**.365**	-.038	**.137**	**-.164**	-	
18. Negative COVID-19 Events	.019	**.086**	.063	.075	**.138**	.046	.057	**.134**	**.103**	**-.092**	-.066	.065	.026	**-.213**	**.183**	**-.261**	**.423**	-
19. Approval of COVID-19 restrictions/behaviors	**.177**	.065	**.104**	.027	**.205**	**.131**	-.072	.113	-.049	**-.138**	**-.093**	-.049	-.014	**-.301**	**.346**	**-.457**	**.324**	**.294**

*Note*. Bold denotes p < .001.

For hesitancy to receive a COVID-19 vaccine, the endorsement rates for the response options to the question, “How likely are you to get a COVID-19 vaccine?”, were: 38.3% Very likely, 30.8% Somewhat likely, 17.0% Somewhat unlikely, and 13.8% Very unlikely. Scores on the anti-vax scale and responses to the COVID-19 vaccine hesitancy item were correlated at *r* = .48, *p* < .001, indicating people with more anti-vax beliefs were also likely to report greater COVID-19 vaccine hesitancy.

Descriptive information for the vaccine outcomes across different demographic groups is presented in Table 5, along with Cohen’s *d*s for the mean differences. Non-White participants (especially Black participants) and people with lower incomes and less education were more likely to endorse anti-vax attitudes, while older participants were less likely to have anti-vax attitudes. Women, non-White participants (especially Black participants), and people with lower incomes and less education reported greater COVID-19 vaccine hesitancy. Participants that were older and reported higher incomes expressed greater willingness to receive a COVID-19 vaccine.

**Table 5:** Descriptive statistics and group differences in vaccine outcomes in fall 2020.

	Anti Vax				COVID-19 Vaccine Hesitancy			
	M	SD	N	*d*	M	SD	N	*d*
Sex								
*Women*	50.2	9.9	513		51.7	10.2	513	
Men	49.7	10.1	491	-.05	48.2	9.5	491	-.37
Race								
*White*	49.1	9.9	764		49.2	9.8	764	
Black	53.8	10.5	144	.46	54.0	10.7	144	.46
Other	51.5	8.8	96	.26	50.3	9.3	96	.11
Age								
*30–59*	51.2	10.1	530		50.8	9.9	530	
Under 30	50.8	11.0	179	-.04	51.8	10.2	179	.09
60 and Older	47.2	8.5	289	-.43	47.5	9.6	289	-.35
Annual Income								
*$50*,*000-$100*,*000*	48.7	9.5	334		49.6	9.9	334	
Below $50,000	52.3	10.4	343	.36	52.8	10.1	343	.32
Above $100,000	48.9	9.7	327	.02	47.5	9.3	327	-.21
Education								
*4-Year College Degree or Higher*	49.1	9.9	572		48.5	9.5	572	
Less than 4-Year College Degree	51.1	10.0	432	.20	52.0	10.3	432	.35

*Note*. M = mean; SD = standard deviation; N = number of respondents; *d* = Cohen’s *d*. Cohen’s *d*s calculated in referent groups; referent groups are *italicized* (Women, White, 30–59 Years Old, Annual Income of $50,000-$100,000, and 4 Year College Degree Or More).

### COVID-19 vaccine hesitancy

Zero-order correlations, standardized regression coefficients, and *R*^2^ values from the multiple regression models predicting COVID-19 vaccine hesitancy are presented in Table 6. The correlations between the demographic variables and COVID-19 vaccine hesitancy were consistent with the mean differences reported above. COVID-19 vaccine hesitancy was unrelated to personality traits, depression, and substance use, and only had a small association with not using social media. COVID-19 vaccine hesitancy had medium to small associations with less liberal social attitudes, more conservative social attitudes, and higher approval of President Trump. COVID-19 vaccine hesitancy had a large association with anti-vax attitudes, less adherence to COVID-19 safety behaviors and lower approval of government restrictions and had small associations with less COVID-19 related stress/worry and experiencing fewer negative impacts associated with the pandemic.

**Table 6:** Standardized regression coefficients from COVID-19 vaccine hesitancy models.

Variable	*r*	Model 1	Model 2	Model 3	Model 4	Model 5
Age	-.16	**-.13 [-.19, -.07] < .001**	**-.17 [-.25, -.09] < .001**	**-.18 [-.26, -.11] < .001**	**-.11 [-.19, -.04] .002**	**-.09 [-.16, -.02] .008**
Men	-.18	**-.08 [-.14, -.02] .012**	**-.09 [-.15, -.02] .015**	**-.10 [-.17, -.04] .002**	**-.12 [-.18, -.05] < .001**	**-.12 [-.18, -.06] < .001**
Non-White	.14	**.15 [.09, .22] < .001**	**.14 [.08, .21] < .001**	**.16 [.10, .22] < .001**	**.18 [.12, .24] < .001**	**.10 [.04, .16] .001**
Household Income	-.23	**-.15 [-.21, -.08] < .001**	**-.16 [-.23, -.10] < .001**	**-.14 [-.21, -.08] < .001**	**-.12 [-.18, -.06] < .001**	**-.08 [-.14, -.02] .007**
Educational Attainment	-.18	**-.08 [-.15, -.02] .016**	**-.08 [-.15, -.01] .023**	-.06 [-.13, .00] .066	-.04 [-.10, .02] .225	-.04 [-.10, .16] .168
Extraversion	-.02		.03 [-.04, .10] .408	.00 [-.07, .07] .990	.00 [-.06, .07] .915	-.01 [-.07, .05] .815
Agreeableness	.01		-.02 [-.10, .06] .597	-.01 [-.08, .06] .779	.02 [-.05, .10] .503	.02 [-.04, .08] .549
Conscientiousness	-.02		.02 [-.06, .10] .721	.00 [-.08, .08] .977	.03 [-.05, .10] .500	.05 [-.02, .12] .167
Negative Emotionality	.04		-.05 [-.15, .04] .250	-.06 [-.15, .03] .211	-.04 [-.12, .05] .386	-.02 [-.09, .06] .677
Openness	-.01		-.05 [-.11, .02] .163	.02 [-.05, .08] .569	.02 [-.04, .08] .484	.01 [-.05, .06] .859
Depression	.02		.00 [-.10, .09] .935	-.02 [-.12, .08] .721	.05 [-.05, .15] .297	.04 [-.06, .13] .450
Binge Drinking	.01		-.04 [-.12, .03] .268	.01 [-.07, .08] .867	-.02 [-.09, .05] .547	-.02 [-.09, .05] .538
Smoking	.05		.04 [-.03, .11] .260	.04 [-.03, .11] .236	.04 [-.03, .10] .276	.01 [-.05, .07] .771
Social Media Use	.11		**.09 [.02, .16] .009**	**.08 [.02, .15] .014**	.06 [.00, .13] .048	.06 [.00, .12] .033
Social Media Problem Use	.01		**-.14 [-.23, -.04] .004**	**-.14 [-.23, -.05] .003**	-.07 [-.16, .02] .143	**-.11 [-.20, -.02]** .014
Conservative Social Attitudes	.19			.02 [-.07, .10] .744	.01 [-.08, .09] .904	-.06 [-.14, .02] .135
Liberal Social Attitudes	-.27			**-.20 [-.28, -.12] < .001**	**-.14 [-.22, -.07] < .001**	**-.10 [-.17, -.02] .009**
Trump Approval	.15			**.12 [.03, .20]** .007	.00 [-.09, .09] .962	-.02 [-.10, .07] .703
COVID-19 Stress/Worry	-.16				-.04 [-.12, .04] .316	-.06 [-.14, .02] .124
Negative COVID-19 Events	-.16				-.04 [-.10, .03] .261	-.01 [-.08, .05] .678
Approval of COVID-19 restrictions and Safety Behaviors	-.37				**-.30 [-.37, -.22] < .001**	**-.22 [-.29, -.14] < .001**
Anti Vax Beliefs	-.48					**.38 [.31, .44] < .001**
R^2^		.10	.12	.19	.27	.36

*Note*. 95% confidence presented in brackets under coefficients; bold denotes that confidence intervals do not include 0. P-values are reported under confidence intervals.

The final model that included all the predictor variables accounted for 36% of the variance in COVID-19 vaccine hesitancy. The largest effects were observed for anti-vax attitudes (*β* = .38) and less adherence to COVID-19 safety behaviors and lower approval of government restrictions (*β* = -.22). We also detected small effects for less social media problem use (*β* = -.11), less liberal social attitudes (*β* = -.10), and demographic variables including younger age (*β* = -.09), female sex (*β* = -.12), non-White race (*β* = .10), and lower income (*β* = -.08). The negative association between vaccine hesitancy and social media problem use seems to be a suppressor effect that emerged after adjusting for the common variance between social media problem use and age (age has significant negative associations with vaccine hesitancy and social media problem use) and anti-vax attitudes (anti-vax attitudes have significant positive associations with vaccine hesitancy and social media problem use), and so is not interpreted further.

### Anti-vax attitudes

Zero-order correlations, standardized regression coefficients, and *R*^2^ values from the multiple regression models predicting anti-vax attitudes are presented in Table 7. The correlations between the demographic predictors and anti-vax attitudes were consistent with the mean differences reported above. Anti-vax attitudes had small but significant associations with low conscientiousness, depression, binge drinking, and nicotine use, and a medium association with social media problem use. Anti-vax attitudes also had medium to large associations with more conservative social attitudes, less liberal social attitudes, and higher approval ratings of President Trump. Finally, anti-vax attitudes were associated with less adherence to COVID-19 safety behaviors, less approval of COVID-19 related government restrictions, and experiencing slightly fewer negative impacts due to the COVID-19 pandemic.

**Table 7:** Standardized regression coefficients from anti vax models.

Variable	*r*	Model 1	Model 2	Model 3	Model 4
Age	-.16	**-.19 [-.25, -.12] < .001**	-.08 [-.15, .00] .052	**-.10 [-.17, -.03] .006**	-.05 [-.12, .02] .148
Men	-.03	**.07 [.01, .13]** .029	.03 [-.04, .10] .037	.01 [-.05, .08] .698	.00 [-.06, .06] .937
Non-White	.16	**.20 [.13, .25] < .001**	**.18 [.12, .24] < .001**	**.19 [.13, .25] < .001**	**.20 [.15, .26] < .001**
Household Income	-.16	**-.12 [-.19, -.05] < .001**	**-.15 [-.21, -.08] < .001**	**-.12 [-.18, -.06] < .001**	**-.11 [-.17, -.05] .001**
Educational Attainment	-.10	-.04 [-.11, .02] .202	-.04 [-.11, .02] .220	.00 [-.07, .06] .887	.01 [-.05, .07] .837
Extraversion	.00		.05 [-.03, .12] .221	.01 [-.06, .08] .772	.01 [-.06, .08] .798
Agreeableness	-.07		-.02 [-.09, .06] .700	-.01 [-.08, .06] .759	.01 [-.06, .08] .725
Conscientiousness	-.15		-.03 [-.11, .05] .412	-.08 [-.15, .00] .048	-.06 [-.13, .02] .140
Negative Emotionality	.07		-.07 [-.16, .03] .181	-.06 [-.15, .03] .169	-.05 [-.14, .03] .218
Openness	-.06		-.06 [-.13, .00] .065	.04 [-.02, .11] .199	.04 [-.02, .10] .181
Depression	.16		.02 [-.07, .11] .669	.04 [-.04, .13] .328	.04 [-.05, .13] .336
Binge Drinking	.18		**.08 [.01, 16] .029**	.02 [-.05, .09] .542	.00 [-.07, .07] .971
Smoking	.19		**.09 [.01, .16] .021**	**.08 [.01, .14] .025**	**.07 [.01, .14] .031**
Social Media Use	.12		.02 [-.05, .09] .521	.01 [-.05, .07] .702	.01 [-.06, .07] .883
Problematic Social Media Use	.24		**.12 [.03, .20] .009**	.08 [.00, .16] .059	**.12 [.03, .20] .006**
Conservative Social Attitudes	.39			**.20 [.12, .27] < .001**	**.18 [.11, .26] < .001**
Liberal Social Attitudes	-.33			**-.16 [-.24, -.09] < .001**	**-.12 [-.20, -.05] .002**
Trump Approval	.28			**.12 [.04, .20] .002**	.05 [-.03, .13] .248
COVID-19 Stress/Worry	-.01				.05 [-.02, .12] .165
Negative COVID-19 Events	-.18				-.07 [-.13, .00] .051
Approval of COVID-19 restrictions and Safety Behaviors	-.33				**-.22 [-.31, -.14] < .001**
R^2^		.08	.14	.28	.32

*Note*. 95% confidence presented in brackets under coefficients; bold denotes that confidence intervals do not include 0. P-values are reported under confidence intervals.

The final regression model that included all the predictors accounted for 32% of the variance in anti-vax attitudes. The largest unique effects were observed for non-White race (*β* = .20), conservative social attitudes (*β* = .18), and less adherence to COVID-19 safety behaviors and less approval of government restrictions (*β* = -.22). We also detected small effects for less liberal social attitudes (*β* = -.12), lower income (*β* = -.11), greater social media problem use (*β* = .12), and greater nicotine use (*β* = .07).

## Discussion

We examined the demographic, psychological, political, and behavioral correlates of anti-vax attitudes and COVID-19 vaccine hesitancy in a national online sample of American adults. We sought to determine the relative predictive power of stable demographic and psychological variables versus factors more proximal to COVID-19 and vaccination itself. We found that younger age, non-White race, lower income, less education, more conservative and less liberal social attitudes, and less adherence to COVID-19 safety behaviors and lower approval of government restrictions were common correlates of anti-vax attitudes in general and COVID-19 vaccine hesitancy specifically.

Younger age was associated with greater vaccine hesitancy, though it was not a significant predictor of anti-vax attitudes in the multiple regression model. Older adults are at greater risk of serious illness and death from COVID-19, which likely accounts for the greater willingness of older participants to receive a COVID-19 vaccine. In contrast, younger people may be more likely to believe that receiving a COVID-19 vaccine is relatively unimportant or would not incur significant benefits [19]. Negative associations between income and vaccine hesitancy are consistent with some prior findings [20]. These associations, however, may be complicated by broader socioeconomic status, which incorporates both income and educational attainment [24]. Indeed, income and educational attainment were moderately correlated in our sample (*r* = .39) and lower educational attainment consistently predicted anti-vax attitudes and COVID-19 vaccine hesitancy. Lower educational attainment may discourage vaccine reception via susceptibility to misinformation and reduced capability to consider the scientific nuances of vaccines [24].

Next, the negative attitudes towards vaccines in non-White participants may be traced to historical and present-day racial injustices and disparities in the American medical establishment. Systematic racial disparities in medical care are widespread as evidenced, for example, in the high maternal and infant mortality rates, bias in pain management, and the disparities in illness and death from COVID-19 in Black Americans [43–46]. Consequently, many Black Americans harbor mistrust of the U.S. medical establishment and thus are suspicious of vaccines [19]. Further, several barriers to medical care are more common among Black Americans including lack of health insurance, high out-of-pocket expenses for things like copays, a history of poor quality of care, etc., all of which may directly affect vaccine uptake [47].

Social and political attitudes had strong associations with anti-vax attitudes and significant but weaker associations with COVID-19 vaccine hesitancy. We found that people who disagreed with relatively liberal social attitudes (e.g., acceptance of homosexuality, women working outside the home) tended not to support vaccination—both in general and specifically for the COVID-19 vaccine. Conservative and authoritarian attitudes were also strongly associated with anti-vax attitudes, though they had a weaker association with COVID-19 vaccine hesitancy. Both liberal and conservative social attitudes were assessed using items from the Right-Wing Authoritarian scale, which conceptualizes the authoritarian personality as having unwavering obedience to a respected leader and the desire for strict societal rules [39]. This desire is often exhibited as a commitment to traditional social values and disdain, resistance, and repression of non-traditional political and social movements. It is surprising, then, that more authoritarian attitudes (i.e., less liberal, more conservative) were associated with ignoring recommendations for vaccinations from medical and political authorities. One explanation may be that political parties most aligned with conservative voters (the Republican party in the United States) have positioned themselves to be in opposition to and undermine the legitimacy of certain authorities that are perceived to be progressive such as academics and scientists, and even some government agencies (e.g., Centers for Disease Control, Food and Drug Administration). These progressive “out groups” are thus deemed less credible than trusted “in group” conservative authorities [8]. In fact, some research has demonstrated conservative social attitudes are associated with a general distrust of medical and scientific experts among conservatives [48].

Finally, anti-vax attitudes and COVID-19 vaccine hesitancy were associated with a lack of adherence to COVID-19 safety behaviors and support for government restrictions to mitigate the spread of COVID-19. In other words, if an individual does not believe that vaccines against COVID-19 and other infectious diseases are necessary and important, then they will not be motivated to participate in related health and safety behaviors. This aligns with recent COVID-19 related literature showing that greater belief in general vaccine effectiveness and/or importance is negatively associated with willingness to receive a COVID-19 vaccine [19]. People with anti-vax attitudes may approach other novel health issues with a similar heuristic structure as well, where one’s opinion about the severity of a disease directly influences their health behavior. More broadly, anti-vax attitudes may be associated with a general distrust of medical advice and intervention especially when endorsed by the government, including the COVID-19 pandemic. This is well understood through the lens of credibility heuristics, i.e., that individual perception of source credibility determines their evaluation of an argument [8].

### Anti-vax attitudes

Anti-vax attitudes were also associated with low conscientiousness, depression, binge drinking, nicotine use, problematic social media use, higher approval of President Trump, and experiencing slightly fewer negative impacts due to the COVID-19 pandemic. We hypothesized that several facets of psychological adjustment might relate to anti-vax attitudes, though it may be more constructive to conceptualize these specific facets as converging onto a broader psychological structure associated with poor health behavior in general. These psychological and behavioral characteristics include the small associations with low conscientiousness, smoking, heavy drinking, and depression. These traits and behaviors may be indicators of a general lack of concern about one’s health and tendency to disregard health recommendations, or they may be psychological characteristics that impair one’s ability to learn about best health practices or the ability to implement changes necessary to experience the benefit of those practices. These associations, however, were small and many were no longer significant after adjusting for other more salient variables (e.g., race, social and political attitudes).

The association between anti-vax attitudes and problematic social media use might also be attributed to poor psychological adjustment in general, as problematic social media use exhibited strong correlations with depression (*r* = .57), binge drinking (*r* = .44), smoking (*r* = .39), and conscientiousness (*r* = -.43). Problematic social media use, however, continued to have an incremental association with anti-vax attitudes after controlling for these psychological variables, suggesting problematic social media use also has a direct effect on the acquisition of anti-vax attitudes, which is reasonable given the effort anti-vax groups have invested in propagating their beliefs through social media platforms [49].

The association between anti-vax attitudes and the approval of President Trump reflects a broader political alignment among conservative interest groups and the rightwing of the modern anti-vax movement. This interpretation is further buttressed by the weaker association between approval for President Trump and COVID-19 vaccine hesitancy, that is, the rightwing of the anti-vax movement fits within a broader conservative political coalition in the United States that pre-dates the COVID-19 pandemic. The small negative association between anti-vax attitudes and experiencing negative impacts of COVID-19 is unclear. For example, characteristics associated with anti-vax and more conservative social attitudes might account for fewer negative events, or people with those beliefs may be engaging in behaviors that reduce the risk for experiencing negative impacts.

### COVID-19 vaccine hesitancy

The only unique predictors of hesitancy for a COVID-19 vaccine were female sex and general anti-vax attitudes. Historically, women have been integral players in the anti-vax movement. Since mothers are disproportionately the primary caregivers of children, they are often the coordinator and final decision maker about children’s medical care. It may follow that women face increased anxieties surrounding the safety of vaccines both for their children—and in the case of COVID-19—themselves.

The connection between general anti-vax attitudes and hesitancy for a COVID-19 vaccine follows from the principle of the general attitude influencing attitudes about a specific case. Consequently, it is important to reflect on the mean-level of anti-vax attitudes within a society and how that will impact an effective societal response that includes the widespread adoption of vaccines to mitigate and potentially eradicate a disease. Based on our sample estimates, over 60% of Americans do not hold anti-vax attitudes while only 10%-15% endorse anti-vax sentiments. This suggests a relatively low ceiling for anti-vax attitudes, but also a notable minority that are unlikely to accept vaccinations under most circumstances and another 25%-30% of Americans who have mixed opinions on anti-vax attitudes. Unfortunately, if a significant minority of the population is unwilling to vaccinate (over 30% in our sample), this significantly reduces the effectiveness of vaccination as a public health intervention to achieve herd immunity and eradicate a disease like COVID-19. The consequence of this is that the disease continues to circulate within the population, infecting the unvaccinated and allowing time for mutations of the virus to occur that could result in variants that are resistant to medical intervention and vaccines. Similarly, having allowed anti-vax attitudes to persist in American society has created conditions that are now contributing to a vulnerability in effectively fighting this and future pandemics.

An understanding of the correlates of anti-vax attitudes beyond COVID-19 could help public health officials develop targeted approaches for certain vaccine-hesitant populations. Several positive public health outcomes can be achieved if anti-vax attitudes are appropriately addressed and curbed, including the eradication of diseases and fewer medical and public health costs. On the other hand, failure to appropriately curb vaccine refusal may incur negative public health consequences. For example, caregivers have been increasingly refusing vaccinations for their kindergarteners, citing personal or moral reasons, which has led to a direct increase in measles cases, which was thought to be eradicated in the United States just 20 years ago [50]. It is possible that COVID-19 may follow a similar trajectory, even if widespread vaccination is achieved within the next few years.

Taken together, these results help explicate factors related to the maintenance of ongoing public health concerns. As COVID-19 cases proliferate while a large percentage of the population remains unvaccinated, this demonstration of the relative predictive power of demographic, trait, and state factors can be utilized to develop educational campaigns and public health interventions.

### Limitations and future directions

While this study provides novel insights into anti-vax attitudes, there were some limitations. Importantly, we were unable to examine effects in non-Black minority groups due to a small sample size. All racial and ethnic disparities related to public health should be a focus of future study. Next, we did not examine the reasons why or why not respondents are willing to receive a COVID-19 vaccine. Thus, we were unable to consider potential barriers to receiving a vaccine, that is, lack of health insurance, transportation issues, and even fear of needles [51]. Some individuals may also have medical issues in which receiving a COVID-19 vaccine would not be advised, for example, an allergy to a vaccine ingredient or compromised immune conditions that increase the risk of adverse reaction to vaccines. Such barriers have the potential to directly influence vaccine reception, whether a person wishes to be vaccinated or not. Additionally, there was substantial variance in both anti-vax attitudes and COVID-19 vaccine hesitancy that was unaccounted for indicating there are other variables that remain to be identified.

Next, we faced inevitable challenges related to reliability and validity for our COVID-19 vaccine hesitancy variable. First, internal consistency cannot be computed with a single-item response. The current study was also cross-sectional thus retest reliability could not be computed. Further, it is difficult to establish convergent and discriminant validity when, to our knowledge, no validated scales of willingness to receive a COVID-19 exist. Therefore, we rely on the face validity of the question and associations with other variables in the data set. For example, the large correlation (*r* = .48) between COVID-19 vaccine hesitancy and our Anti-Vax attitudes scale.

The most important limitation is that the COVID-19 pandemic is an ongoing and dynamic situation. Most relevant to the current analyses was that a vaccine was not yet available when this data was collected. Further, a presidential election also occurred that resulted in a shift of power from a conservative (aligned with anti-vax advocates) to a liberal political party. These events may have led to substantial changes in attitudes regarding vaccination. Future work will follow up with respondents over the course of the pandemic and post-pandemic to determine the trajectory of anti-vax attitudes and the long-term health impacts of vaccine hesitancy.
